# Natural and Synthetic Compounds, Swords for Glioblastoma Therapy: From Tumor to Its Microenvironment

**DOI:** 10.3390/ijms27177798

**Published:** 2026-08-31

**Authors:** Bingxia Huang, Yan Wang

**Affiliations:** 1 Engineering Research Center of Tropical Medicine Innovation and Transformation of Ministry of Education, Hainan Key Laboratory for Research and Development of Tropical Herbs, Haikou Key Laboratory of Li Nationality Medicine, School of Pharmacy, Hainan Medical University, Haikou 571199, China; 18212194387@163.com; 2 Hainan Academy of Medical Sciences, Hainan Medical University, Haikou 571199, China

**Keywords:** glioblastoma, small-molecule compounds, tumor microenvironment, immune regulation, drug resistance

## Abstract

Glioblastoma (GBM) is the most aggressive primary brain malignancy in adults, which remains difficult to treat because of extensive intratumoral heterogeneity, intrinsic and acquired treatment resistance, a profoundly immunosuppressive tumor microenvironment (TME), and restricted drug delivery across the blood–brain barrier (BBB). Owing to their relatively low molecular mass, potential for BBB penetration, and ability to modulate multiple targets, natural and synthetic compounds have attracted increasing interest as candidates for GBM treatment. This narrative review summarizes the mechanisms by which naturally derived compounds—including saponins, flavonoids, and sesquiterpene lactones—and synthetic small molecules exert anti-GBM effects on tumor and the TME. Their reported actions include suppressing key prosurvival pathways, such as the phosphoinositide 3-kinase/protein kinase B/mechanistic target of rapamycin (PI3K/AKT/mTOR), nuclear factor kappa B (NF-κB), and mutant p53 signaling; activating regulated cell-death processes, including apoptosis, pyroptosis, and parthanatos, as well as autophagy-associated cell death; and remodeling the tumor immune milieu to promote CD8^+^ T-cell infiltration. In preclinical models, some of these agents also overcome temozolomide (TMZ) resistance and resensitize glioma stem cells (GSCs) to chemotherapy or radiotherapy. Future studies should prioritize molecularly informed patient stratification, rational combination strategies, and advanced nanocarrier-mediated delivery platforms to facilitate the clinical translation of small-molecule therapeutics for GBM.

## 1. Introduction

Glioblastoma (GBM) is the most common and aggressive primary malignant brain tumor in adults, whose annual incidence is approximately 3–5 cases per 100,000 population, with the highest burden among individuals aged 45–70 years [1,2]. Under the current World Health Organization (WHO) classification of tumor of the central nervous system (CNS), GBM is defined as an adult-type diffuse astrocytic glioma, isocitrate dehydrogenase (IDH)-wild-type, that meets specific pathological or molecular criteria (microvascular proliferation, necrosis, telomerase reverse transcriptase (TERT) promoter mutation, epidermal growth factor receptor (EGFR) amplification, or +7/−10 chromosomal alterations) and is classified as CNS WHO grade 4 [3]. The current standard of care follows the Stupp protocol, which combines maximal safe surgical resection with postoperative radiotherapy and concomitant temozolomide (TMZ), followed by adjuvant TMZ. Nevertheless, outcomes remain poor: median overall survival (OS) is only 14–16 months, and fewer than 5% of patients survive for five years [3,4]. A barrier to first-line therapy is TMZ resistance, which is predominantly dictated by the O^6^-methylguanine-DNA methyltransferase (*MGMT*) promoter methylation status. Patients with *MGMT* promoter methylation achieve a median OS of ~18.2 months, whereas unmethylated tumors retain robust DNA repair and show TMZ resistance rates exceeding 50% [5]. For recurrent or progressive GBM where Stupp-regimen failure has already occurred, anti-vascular endothelial growth factor (VEGF)-A monoclonal antibody bevacizumab is one of the most frequently used salvage options to reduce peritumoral edema, lower corticosteroid dependence, ameliorate neurological symptoms, and prolong progression-free survival (PFS). However, it confers no OS benefit (16.8 vs. 16.7 months) [6,7], and over 57% of patients exhibit a highly aggressive, diffuse infiltrative progression pattern following bevacizumab treatment [8]. More seriously, combinations of bevacizumab with other targeted agents or chemotherapies have not only failed to improve OS but have exacerbated anemia, headache, and epistaxis, further deteriorating the patients’ quality of life [9,10].

The limited efficacy of existing interventions can be attributed to four principal barriers to GBM eradication: extensive intratumoral heterogeneity, inadequate drug penetration across the blood–brain barrier (BBB), intrinsic and treatment-induced resistance mediated by glioma stem cells (GSCs), and a profoundly immunosuppressive tumor microenvironment (TME) [11,12,13]. To navigate these multifaceted challenges, natural and synthetic compounds have emerged as a cornerstone of innovative anti-GBM drug discovery. Unlike macromolecular biologics (e.g., monoclonal antibodies), these compounds possess optimal lipophilicity and structural compactness, facilitating efficient BBB traversal, oral bioavailability, and lower production costs [12]. Crucially, natural and synthetic compounds—spanning highly selective synthetic kinase inhibitors to multi-target naturally derived scaffolds—can disrupt various malignant phenotypes simultaneously. By orchestrating a tripartite regulation of signaling networks, cell death modalities, and the immune microenvironment, small molecules can effectively bypass the compensatory drug resistance pathways commonly associated with single-target therapies [14]. This review systematically elucidates the primary molecular mechanisms, evaluates representative compounds and their clinical progress, and outlines future strategic directions for small-molecule therapeutics in GBM.

## 2. Methods

This narrative review was conducted through a comprehensive literature search of the PubMed, Web of Science, Scopus, and Google Scholar databases. Relevant publications were identified, from database inception to June 2026, with particular emphasis on studies published between September 2024 and June 2026 to incorporate the most recent advances in glioblastoma (GBM) research. The final literature search was completed on 30 June 2026. The literature search strategy was adapted from a previously described approach for narrative reviews [15], with further refinement using MeSH terms, free-text terms, and the Boolean operators “AND” and “OR”. Representative search terms included glioblastoma, glioblastoma multiforme, small molecules, natural products, synthetic compounds, BBB, tumor microenvironment, drug resistance, immune modulation, cell death, PI3K/AKT/mTOR, NF-κB, p53, and clinical translation.

Eligible publications included original preclinical and clinical studies, as well as high-quality review articles and meta-analyses that provided important mechanistic, epidemiological, or clinical backgrounds. The mechanistic conclusions presented in this review were primarily based on original experimental studies, whereas review articles were used to summarize established concepts and provide contextual information.

Publications were screened according to their relevance to the molecular mechanisms, therapeutic targets, and translational applications of naturally derived compounds and synthetic products in GBM. Articles lacking sufficient experimental evidence, those unrelated to GBM biology or therapy, conference abstracts, editorials, and duplicate publications were excluded.

## 3. Molecular Characteristics of GBM and Potential Therapeutic Targets

On the basis of transcriptomic characteristics, GBM has historically been stratified into four molecular subtypes: proneural, classical, mesenchymal, and neural [16]. The proneural subtype commonly exhibits alterations in platelet-derived growth factor receptor alpha (*PDGFRA*) or *IDH1,* together with enrichment of oligodendrocyte-associated genes, and has generally been associated with comparatively favorable outcomes. The classical subtype is characterized by *EGFR* amplification, cyclin-dependent kinase inhibitor 2A (*CDKN2A*) loss, and wild-type tumor protein p53 (*TP53*). Patients with this subtype may derive substantial benefit from radiotherapy combined with TMZ. By contrast, the mesenchymal subtype frequently harbors neurofibromin 1 (*NF1*) or phosphatase and tensin homolog (*PTEN*) alterations and shows increased expression of mesenchymal markers, extensive necrosis, and pronounced inflammatory-cell infiltration. This subtype may also respond to intensified treatment. The neural subtype is associated with abundant neuronal-marker expression, although this profile may partly reflect contamination by nonmalignant neural cells; its biological and prognostic relevance therefore remains uncertain [16,17].

**Figure 1:**
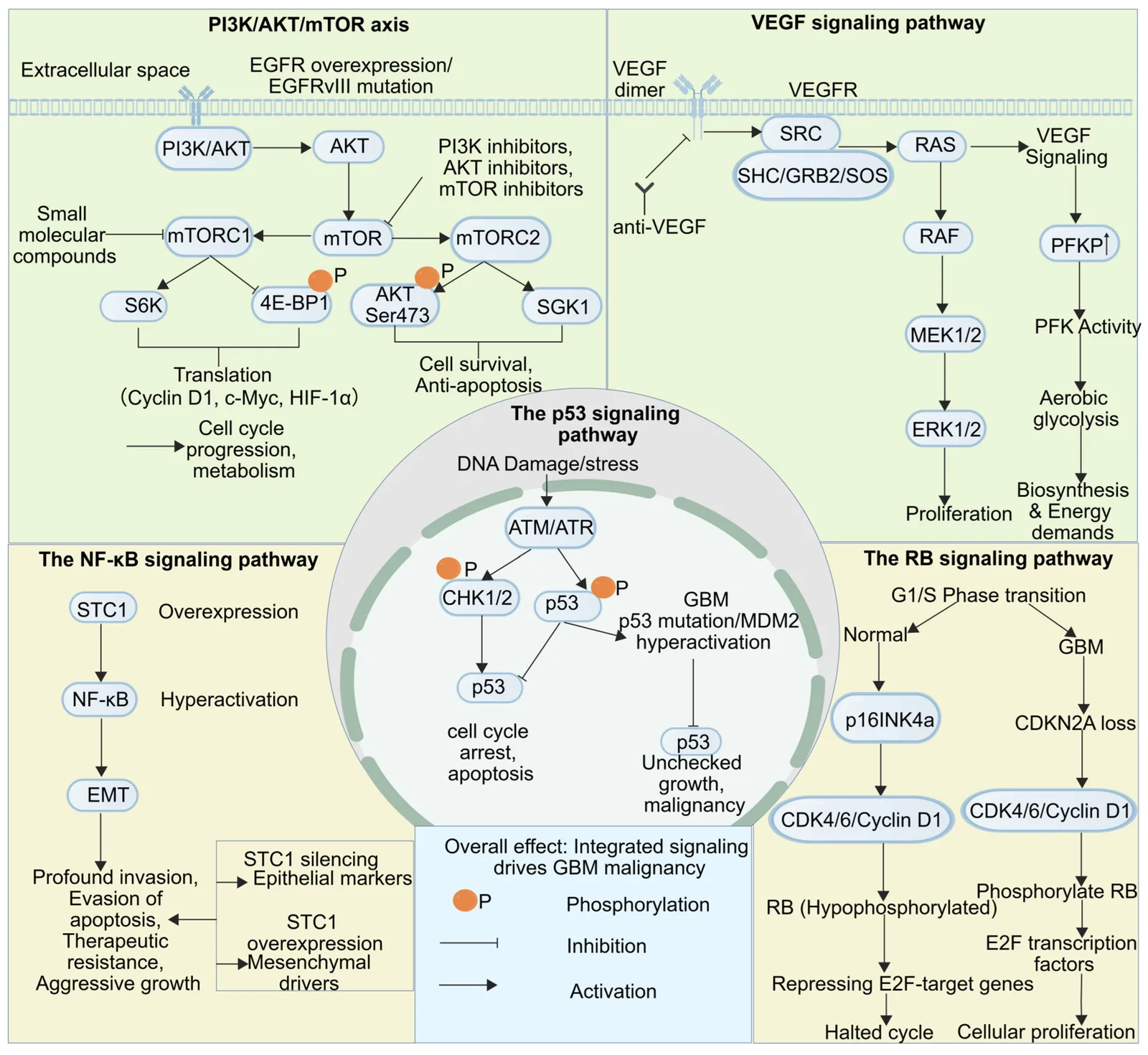
Core molecular signaling pathways driving GBM malignancy and therapeutic resistance. The figure highlights the PI3K/AKT/mTOR, VEGF, p53, NF-κB, and RB signaling pathways.

At the molecular level, GBM development, progression, and treatment resistance are closely associated with dysregulation of several signaling networks, including phosphoinositide 3-kinase (PI3K)/protein kinase B (AKT)/mechanistic target of rapamycin (mTOR), VEGF, p53, nuclear factor kappa B (NF-κB), and retinoblastoma (RB) signaling, all of which represent potential targets for natural and synthetic products (Figure 1).

### 3.1. PI3K/AKT/mTOR Axis

Aberrant EGFR overexpression or oncogenic mutation, exemplified by the *EGFRvIII* variant, occurs in approximately 50% of patients with GBM [18,19]. Persistent EGFR activity engages the downstream PI3K/AKT/mTOR cascade [20], thereby enhancing tumor-cell proliferation and preserving stem-like properties. As a pivotal coordinator of cellular metabolism and mitogenic signaling, mTOR acts through two structurally and functionally distinct assemblies: mTOR complex 1 (mTORC1) and mTOR complex 2 (mTORC2). Nutrient availability is communicated to the mTOR network through Rag-family GTPases. mTORC1 activates the phosphorylation of S6 kinase (S6K) and eukaryotic translation initiation factor 4E-binding protein 1 (4E-BP1), consequently promoting the synthesis of proteins involved in cell-cycle regulation and tumor growth, including cyclin D1, c-Myc, and hypoxia-inducible factor 1-alpha (HIF-1α). In parallel, mTORC2 supports cellular survival and inhibits apoptotic signaling by phosphorylation of AKT at Ser473 to achieve its complete activation and by stimulating serum/glucocorticoid-regulated kinase 1 (SGK1) [21].

### 3.2. VEGF Signaling Pathway

VEGF, historically termed vascular permeability factor (VPF), is a dimeric glycoprotein that functions as a major driver of angiogenesis by engaging VEGF receptors (VEGFRs) on the cell surface. Ligand–receptor binding stimulates intracellular SRC-family tyrosine kinase signaling and recruits the SHC/GRB2/SOS adaptor complex, which promotes activation of the small GTPase RAS. Activated RAS subsequently initiates the classical mitogen-activated protein kinase (MAPK) cascade through activation of RAF, MAPK/ERK kinase 1/2 (MEK1/2), and extracellular signal-regulated kinase 1/2 (ERK1/2). This signaling sequence ultimately enhances malignant-cell proliferation. In addition to supporting survival and growth, VEGF reshapes tumor-cell metabolism by increasing the expression of platelet-type phosphofructokinase (PFKP) and, consequently, elevating phosphofructokinase activity. The resulting metabolic shift intensifies aerobic glycolysis, commonly referred to as the Warburg effect, thereby supplying the energy and biosynthetic substrates required for the rapid expansion of GBM cells [22].

### 3.3. The p53 Signaling Pathway

The tumor-suppressive protein *p53* plays a fundamental role in preserving genome stability and limiting malignant transformation [23]. DNA lesions caused by radiotherapy, chemotherapeutic agents, or stress within the tumor activate damage-sensing kinases, particularly ataxia-telangiectasia mutated (ATM) and ATM- and Rad3-related (ATR). These kinases initiate the downstream checkpoint response by phosphorylating checkpoint kinase 1/2 (CHK1/2) and promoting p53 stabilization, ultimately restraining the proliferation of GBM cells. In most GBM cases, however, the tumor-suppressive activity of p53 is compromised by either direct genetic mutations or excessive activation of its negative regulator, mouse double minute 2 homolog (MDM2). Disruption of this regulatory axis permits uncontrolled tumor-cell expansion and facilitates malignant disease progression [24,25,26].

### 3.4. The NF-κB Signaling Pathway

Constitutive dysregulation of NF-κB signaling represents a prominent molecular feature of GBM. Sustained activation of this pathway contributes to tumor initiation and progression by promoting rapid cellular expansion, extensive infiltration into surrounding brain tissue, resistance to apoptotic death, and reduced therapeutic responsiveness [27]. One important effect of NF-κB activity in GBM is the initiation of an epithelial-to-mesenchymal transition (EMT)-like phenotypic program [28]. Stanniocalcin 1 (STC1), an upstream modulator of this pathway, participates in regulating such cellular plasticity. STC1 knockdown attenuates NF-κB signaling, increases the expression of epithelial-associated proteins such as E-cadherin and occludin, and simultaneously reduces the levels of mesenchymal regulators, including N-cadherin, snail1, and vimentin [29]. In contrast, elevated STC1 expression activates the NF-κB axis and markedly increases cell proliferation in vitro, while also strengthening the capacity of these cells to infiltrate adjacent tissues—a behavior distinct from conventional distant metastasis. Evidence obtained from in vivo xenograft experiments further demonstrates that excessive STC1/NF-κB activity promotes GBM progression and enhances local tumor invasion [29].

### 3.5. The RB Signaling Pathway

The RB signaling pathway serves as a major checkpoint governing cell-cycle progression from G1 into S phase and depends on coordinated interactions among CDKN2A/p16INK4a, cyclin D1, cyclin-dependent kinase 4/6 (CDK4/6), members of the RB protein family—including RB, p107, and p130—and downstream transcriptional regulators [30]. In normal cells, the tumor suppressor p16INK4a directly associates with CDK4/6 and inhibits the assembly and activity of cyclin D1–CDK4/6 complexes. Consequently, RB phosphorylation is restricted, allowing active RB-family proteins to suppress E2 transcription factor (E2F)-dependent transcription. This regulatory mechanism limits the expression of genes required for DNA synthesis and nucleotide production, thereby arresting further cell-cycle progression [31]. In GBM, however, *CDKN2A* is frequently deleted or functionally inactivated. Loss of this inhibitory checkpoint permits persistent RB phosphorylation, sustained E2F activity, and uncontrolled tumor-cell proliferation [32,33].

## 4. Naturally Derived Compounds and Potential Targets

**Figure 2:**
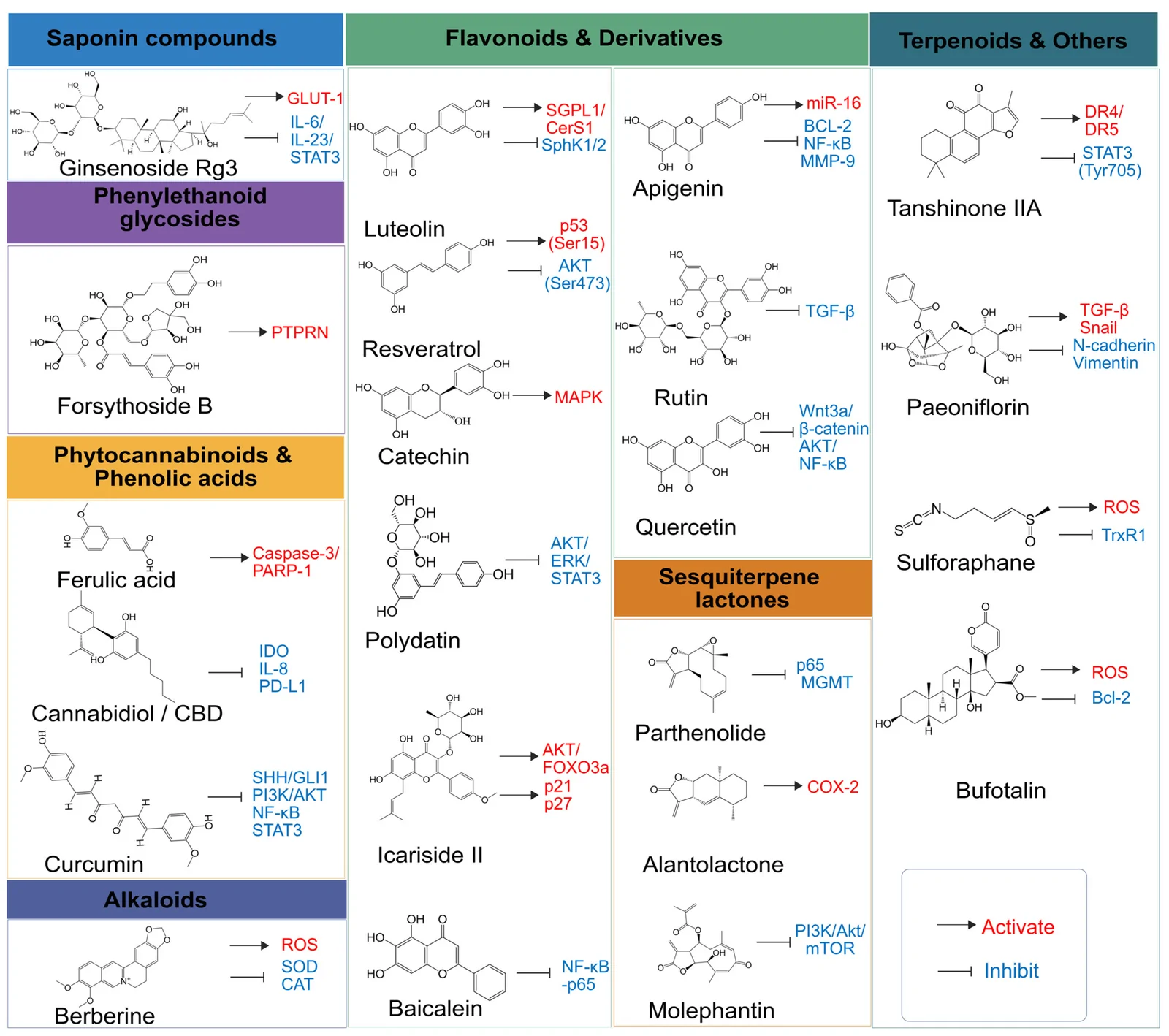
Chemical structures and primary molecular targets of representative natural compounds. The compounds are grouped into saponins, flavonoids and their derivatives, sesquiterpene lactones, phenylethanoid glycosides, phytocannabinoids and phenolic acids, alkaloids, and other terpenoids.

Natural products and structurally optimized analogues constitute a rich source of lead compounds for anticancer drug development and have contributed to the discovery of more than 60% of currently approved chemotherapeutic agents [34]. In the context of GBM, numerous naturally occurring products—including saponins, flavonoids, sesquiterpene lactones, and alkaloids—have displayed substantial antitumor effects through the simultaneous modulation of multiple molecular targets (Table 1 and Table 2, and Figure 2). Notably, the reported anti-GBM potency of these natural products varies substantially across compounds and experimental systems, with active concentrations ranging from 0.1 to 440 μM and showing favorable selectivity toward malignant cells relative to normal astrocytes.

### 4.1. Saponin Compounds: Glucose Transporter 1 (GLUT1)-Mediated Transport and Immune Microenvironment Remodeling

Ginsenoside Rg3 is a dammarane-type saponin with a tetracyclic triterpenoid structure that is predominantly isolated from red ginseng. Its glucose moiety can be recognized as a substrate by GLUT1, thereby promoting carrier-mediated passage through the BBB and preferential enrichment within glioma tissue [65]. In addition to cooperating with paclitaxel to induce apoptotic cell death [65], Rg3 substantially remodels the tumor immune milieu. Mechanistically, it shifts tumor-associated macrophages (TAMs) from the tumor-supportive M2-like state toward the tumoricidal M1-like phenotype, enhances infiltration by CD8^+^ T cells, and reduces the abundance of regulatory T cells (Tregs) and myeloid-derived suppressor cells (MDSCs). Additionally, Zhu et al. revealed that Rg3 extends median survival in in vivo models by inhibiting the interleukin-6 (IL-6)/interleukin-23 (IL-23)/signal transducer and activator of transcription 3 (STAT3) axis [35].

### 4.2. Flavonoids and Their Derivatives: Multitarget Signaling Modulation and Metabolic Rewiring

Flavonoids are polyphenolic compounds whose investigation has expanded beyond the conventional emphasis on antioxidant activity toward a more detailed understanding of their capacity to regulate intracellular signaling circuits [66,67].

#### 4.2.1. Modulators of Sphingolipid Metabolism and Stemness

Luteolin exhibits pronounced activity against TMZ-resistant GSCs carrying an unmethylated MGMT promoter. At concentrations of 10.0–50.0 μM, luteolin inhibits sphingosine kinase 1/2 (SphK1/2) while activating sphingosine-1-phosphate lyase 1 (SGPL1) and ceramide synthase 1 (CerS1). These coordinated effects counteract the growth-promoting activity of exogenously supplied sphingosine-1-phosphate (S1P) and shift sphingolipid metabolism toward the accumulation of proapoptotic ceramide. In addition, studies revealed that resveratrol at 5.0–50.0 μM diminishes GSC stem-like properties by suppressing AKT phosphorylation at Ser473 [37] and increasing p53 activation through phosphorylation at Ser15. Together, these events promote proteasome-dependent degradation of Nanog, a central pluripotency regulator, and subsequently drive GSCs toward terminal differentiation [38].

#### 4.2.2. Regulators of Apoptosis and the Cell Cycle

Zhu et al. revealed that catechin at 12.5–200.0 μM suppresses cell cycle by regulating MAPK/ERK signaling [68], while polydatin, a glycosylated derivative of resveratrol, induces apoptosis by inhibiting EGFR and its downstream AKT, ERK, and STAT3 pathways at concentrations of 150–600 μM [41]. Icariside II exerts anti-GBM activity with an IC_50_ of 30.0 μM by modulating the AKT/forkhead box O3a (FOXO3a) axis. It promotes FOXO3a nuclear translocation and increases the activity of the cyclin-dependent kinase inhibitors p21 and p27, thereby inducing G1-phase arrest and mitochondrial apoptosis [42]. Baicalein, with an IC_50_ of 25.0 μM, induces apoptosis by preventing nuclear accumulation of the NF-κB p65 subunit and shifting the BCL-2/BAX ratio toward apoptosis [43]. Apigenin acts through an epigenetic mechanism by increasing tumor-suppressive microRNA-16 (miR-16) expression. This response suppresses the anti-apoptotic protein BCL-2 and inhibits the NF-κB/matrix metalloproteinase-9 (MMP-9) axis associated with tumor growth [44].

#### 4.2.3. Microenvironment and DNA Repair Inhibitors

Rutin suppresses GBM through two complementary mechanisms. In addition to exerting direct cytotoxic effects on tumor cells, it increases microglia chemotaxis by upregulation of interleukin-1 beta (IL-1β) and tumor necrosis factor (TNF), while reducing transforming growth factor beta (TGF-β) expression, thereby creating a proinflammatory microenvironment that restricts tumor-cell migration in vivo [45]. By comparison, quercetin impairs DNA repair capacity in MGMT-positive GBM models through simultaneous inhibition of the Wnt3a/β-catenin and AKT/NF-κB pathways [46].

### 4.3. Sesquiterpene Lactones: NF-κB Suppression, Intracellular Reactive Oxygen Species (ROS) Generation, and Autophagic Blockade

Sesquiterpene lactones are structurally distinct natural terpenoids characterized by an α-methylene-γ-lactone moiety. This electrophilic group readily forms covalent adducts with nucleophilic sites, including cysteine residues in key protein kinases. In GBM, their reported antitumor activity is principally associated with disruption of NF-κB signaling and increased intracellular oxidative stress [68].

#### 4.3.1. Targeting the NF-κB Axis and Overcoming Chemoresistance

The mesenchymal subtype of GBM displays extensive inflammatory-cell infiltration, pronounced necrosis, and elevated expression of mesenchymal markers, features that are closely associated with persistent NF-κB activity [69]. Sesquiterpene lactones may offer particular therapeutic value for this highly aggressive molecular phenotype. Parthenolide, at a concentration of 5.0 μM, attenuates mesenchymal characteristics by markedly inhibiting phosphorylation of the NF-κB p65 subunit [50] and reducing expression of the DNA repair enzyme MGMT [49]. These effects restore the responsiveness of highly resistant GSCs to TMZ. ACT001, a parthenolide derivative with an IC_50_ of 15.9 μM, broadens this therapeutic strategy by targeting adipocyte enhancer-binding protein 1 (AEBP1) and inhibiting downstream PI3K/AKT signaling, consequently disrupting GSC self-renewal [48]. Alantolactone exhibits an IC_50_ of 16.0 μM and interacts with the Lys147 residue of IκB kinase beta (IKKβ), competitively suppressing its kinase activity. The resulting inhibition prevents IκB degradation and blocks p65 translocation into the nucleus, thereby decreasing the expression of inflammatory mediators such as cyclooxygenase-2 (COX-2). Moreover, its ability to cross the BBB, together with demonstrated in vivo activity and the absence of substantial systemic toxicity, supports the therapeutic potential of alantolactone for GBM [51].

#### 4.3.2. ROS-Mediated Mitochondrial Dysfunction and Autophagy Blockade

Molephantin (EM-5), a natural compound obtained from *Elephantopus mollis*, exerts antitumor activity through several interconnected mechanisms dominated by oxidative stress, with an IC_50_ of 10.6–22.6 μM. EM-5 induces pronounced accumulation of ROS, resulting in loss of mitochondrial membrane potential and oxidative phosphorylation disruption [52]. It also compromises cytoprotective autophagy by blocking the fusion of autophagosomes with lysosomes, thereby preventing completion of autophagic flux and weakening the metabolic adaptability of tumor cells. Through the combined impairment of mitochondrial energy production and autophagy-dependent survival, together with ROS-mediated suppression of the PI3K/AKT/mTOR pathway, EM-5 demonstrates substantial in vivo antitumor activity and favorable permeability across the BBB [52].

### 4.4. Phenylethanoid Glycosides: Epigenetic Target Restoration and Cell Cycle Arrest

Forsythoside B, a natural compound isolated from *Forsythia suspensa*, acts through a distinctive epigenetic-like regulatory mechanism involving marked upregulation of protein tyrosine phosphatase receptor type N (PTPRN). Higher *PTPRN* expression has been associated with lower WHO tumor grades and longer OS in patients with glioma [70]. Restoration of PTPRN expression by forsythoside B induces G0/G1-phase arrest and apoptotic death in vitro. Although the IC_50_ of forsythoside B in vitro is approximately 200.0 mM, forsythoside B reprograms the TME toward an antitumor immune state in orthotopic tumor models [53]. Such a high effective concentration substantially limits direct clinical translation because of potential off-target toxicity and physiologically unattainable solubility or exposure. Future GBM drug-development efforts should therefore prioritize the identification and optimization of more potent PTPRN-targeting derivatives.

### 4.5. Alkaloids: ROS-Mediated Bioenergetic Disruption and Cell Cycle Blockade

Alkaloids comprise a chemically heterogeneous family of natural organic molecules containing one or more nitrogen atoms. Their anti-GBM activity involves interference with tumor-cell bioenergetics and modulation of the immune microenvironment. Berberine, a representative isoquinoline alkaloid, induces G1-phase cell-cycle arrest and promotes early apoptotic death in vitro, with the apoptotic fraction reaching 53.5% at a concentration of 25.0 μM. This effect is mechanistically associated with perturbed oxidative homeostasis, including substantial intracellular accumulation of ROS, an increase in thiobarbituric acid reactive substances (TBARSs), and carbonylation [54].

### 4.6. Phytocannabinoids: Dual Cytotoxicity, Antiangiogenic, and Immune Checkpoint Inhibition

Cannabidiol (CBD) combines direct tumor-cell toxicity with substantial indirect immunomodulatory activity. Its potency varies among GBM models, with reported IC_50_ values of 1.5 μM in T98G cells, 17.4 μM in U87MG cells [56], and 3.5 μM in GSCs [71]. CBD induces glioma cell death through multiple regulated cell-death pathways, including ferroptosis, autophagy, and apoptosis. Mechanistically, CBD activates ERK signaling (without affecting JNK and p38 pathways), elevates ROS release, endoplasmic reticulum stress, and intracellular iron accumulation, and reduces glutathione (GSH) levels. Correspondingly, CBD upregulates autophagy-related proteins (LC3-II, Atg7, Beclin-1) and modulates ferroptosis-associated proteins, including glutathione peroxidase 4 (GPX4), solute carrier family 7 (anionic amino-acid transporter light chain), member 11 (SLC7A11), and TFRC [72]. Furthermore, CBD affects the dynamics of the TME by repressing P-selectin, apelin, and interleukin-8 (IL-8) and blocking the key immune checkpoint protein–indoleamine 2,3-dioxygenase (IDO) to promote antitumor immunity and facilitate CD8^+^ T-cell infiltration [55]. Notably, Khodadadi et al. reported that prophylactic inhalation of CBD at 10 mg/day for 14 days markedly reduced SOX2-positive stem-like cells in orthotopic models. This intervention also decreased the expression of the immune-evasion molecules IDO and programmed death-ligand 1 (PD-L1), as well as the chemoresistance-associated enzyme MGMT [56].

### 4.7. Terpenoids and Others: Receptor Modulation, EMT Inhibition, and Mitochondrial Catastrophe

This group comprises chemically heterogeneous molecules, including phenanthrenequinones, isothiocyanates, bufadienolides, monoterpene glycosides, and compounds containing a mono-tetrahydrofuran (mono-THF) ring. Zhou et al. showed that tanshinone IIA at 10.0 μM potentiates apoptosis induced by tumor necrosis factor-related apoptosis-inducing ligand (TRAIL). This synergistic response is mediated by death receptors 4 and 5 (DR4 and DR5) overexpression and concurrent inhibition of STAT3 phosphorylation at Tyr705 [56,57]. Paeoniflorin (PF), a polyphenolic compound derived from *Radix paeoniae* Alba, reduces GBM-cell migration and invasion by inhibiting TGF-β signaling and decreasing the expression of EMT markers, including snail, N-cadherin, and vimentin, as well as matrix metalloproteinases. Oral treatment at 1 g/kg/day effectively suppressed the growth of U87MG xenografts, with favorable systemic tolerability and penetration across the BBB [58]. Additionally, phenolic acids such as ferulic acid show anti-GBM effects at a concentration of 36 μM by modulating transglutaminase 2 (TG2) activity [59].

Sulforaphane (SFN), an isothiocyanate compound, irreversibly inhibits thioredoxin reductase 1 (TrxR1), leading to lethal accumulation of mitochondria-derived ROS. SFN also eliminates CD133^+^ GSCs and promotes the polarization of TAMs toward the M1-like phenotype [60,61]. Bufotalin, a member of the bufadienolide family, displays potent cytotoxicity at nanomolar concentrations, with an IC_50_ of 0.1 μM, attenuates EMT progression, and induces extensive mitochondrial ROS production. The resulting oxidative injury decreases BCL-2 expression, suppresses AKT signaling, and acts synergistically with TMZ to inhibit GBM xenograft growth [62].

## 5. Synthetic Compounds and Their Potential Targets

**Figure 3:**
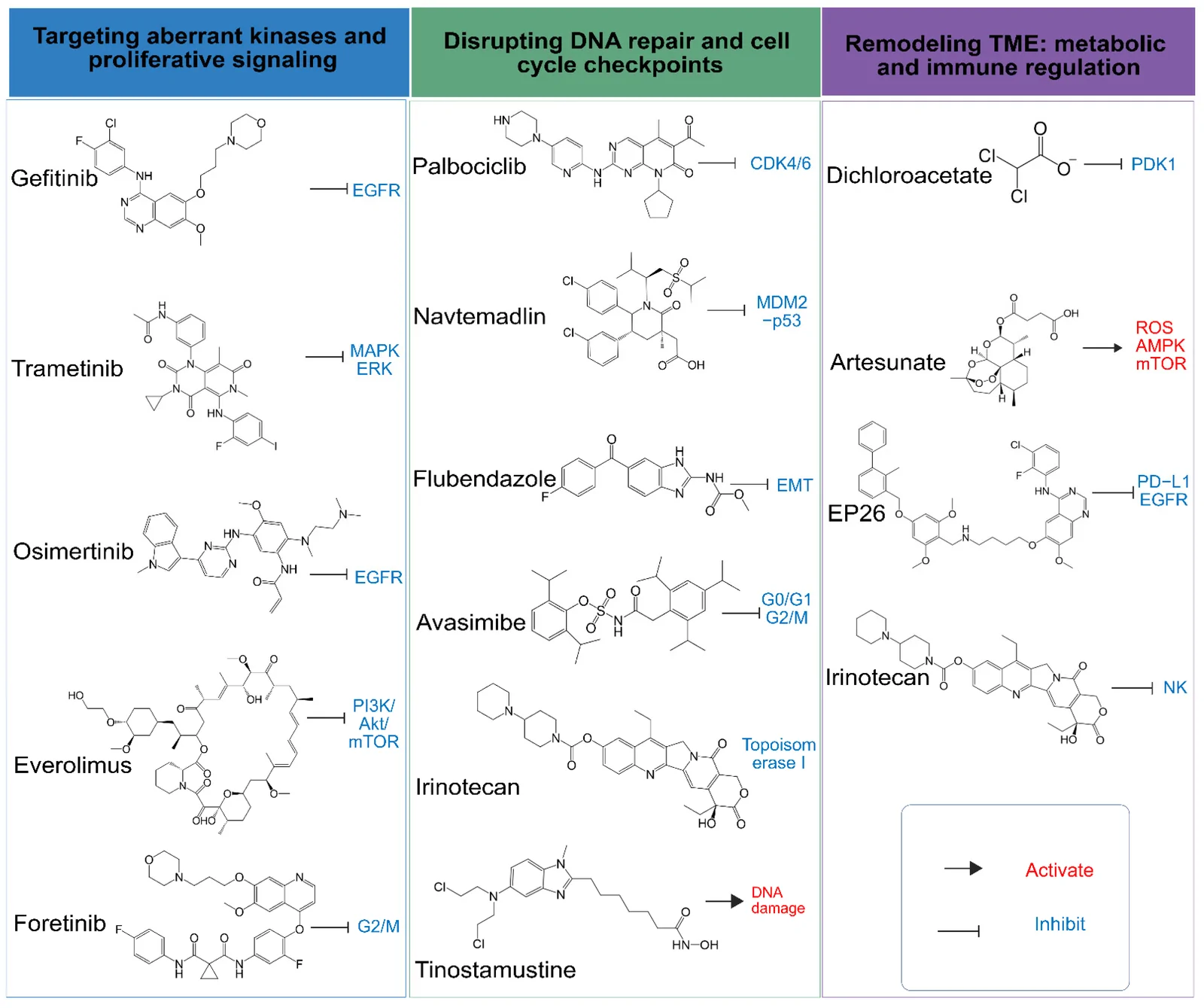
Mechanistic classification and target mapping of synthetic small-molecule compounds in GBM therapy. The compounds are grouped into three categories: agents targeting aberrant kinases and proliferative signaling; agents disrupting DNA repair and cell-cycle checkpoints; and agents remodeling the TME through metabolic or immune regulation.

In contrast to natural molecular scaffolds, which often influence multiple biological targets, synthetic small molecules are typically designed to achieve strong binding affinity and high selectivity for specific kinases. Such target specificity may nevertheless limit therapeutic durability in a molecularly heterogeneous malignancy such as GBM, because tumor cells can rapidly activate compensatory signaling circuits and develop adaptive resistance. Accordingly, contemporary development strategies for synthetic agents increasingly integrate selective pathway blockade with rational drug combinations, epigenetic modulation, and reprogramming of the tumor microenvironment (Table 3 and Figure 3).

### 5.1. Targeting Aberrant Kinases and Proliferative Signaling

Aberrant activation of receptor tyrosine kinases (RTKs) and their downstream signaling networks is a major contributor to GBM progression [89]. This alteration is particularly relevant to the historically defined classical subtype, which frequently exhibits EGFR amplification. However, adaptive signaling responses often limit the durability of RTK-targeted monotherapy. Gefitinib, a first-generation EGFR inhibitor, has an IC_50_ of 20.0 μM in U87MG and U373 cells. However, gefitinib induces an LRIG2/AXL-dependent escape pathway to promote resistance; simultaneous AXL blockade overcomes this resistance and restores antitumor efficacy in vivo [73]. Osimertinib, a third-generation EGFR inhibitor, has an IC_50_ of 4.0–7.0 μM in LN-18, LN-229, U87MG, and SF-539 cells within 24–72 h. Mechanistically, osimertinib targets the protein kinase R-like endoplasmic reticulum kinase (PERK)–eukaryotic translation initiation factor 2 alpha (eIF2α) stress pathway and induces paraptosis-like cell death; however, it induces thyroid hormone receptor interactor 13 (TRIP13) to confer resistance. Hu et al. revealed that TRIP13 levels downregulated by an AKT inhibitor, MK2206, improved the anti-GBM effects of osimertinib in vitro and in vivo [74]. Trametinib, a mitogen-activated protein MEK1/2 inhibitor with an IC_50_ of 0.3 μM in E57 cells, increases phosphorylated focal adhesion kinase (p-FAK) to induce resistance. Combining trametinib with a focal adhesion kinase (FAK) inhibitor suppresses this adaptive activation and promotes apoptosis [75], illustrating that multi-kinase inhibitors provide another strategy for limiting signaling plasticity. Foretinib, which displays IC_50_ values of 4.7–30.0 μM in T98G and U87MG cells, primarily inhibits c-MET phosphorylation, consequently inducing G2/M-phase arrest and suppressing EMT [76]. Nanoparticle-based delivery may also improve drug penetration. Everolimus, a selective mTORC1 inhibitor with IC_50_ values of 0.12–0.45 μM in primary GBM models such as GBM22 and GBM1A, shows improved delivery and antiproliferative effects when incorporated into tumor-targeted liposomal nanoparticles [90].

### 5.2. Disrupting DNA Repair and Cell-Cycle Checkpoints

Therapeutically compromising DNA repair systems and cell-cycle checkpoints represents a promising approach to limiting GBM-cell survival. Among conventional alkylating drugs, the “dual-alkylator” regimen combining TMZ with lomustine, a bifunctional nitrosourea, is applied to patients with newly diagnosed GBM and prolongs median OS from 31.4 to 48.1 months [91]. TMZ reduces the intracellular pool of MGMT, thereby increasing the capacity of lomustine to generate lethal DNA crosslinks [4]. Nimustine, another member of the nitrosourea family, produces DNA double-strand breaks independently of *MGMT* promoter methylation with an IC50 of 260–406 μM in various GBM cells and provides a salvage option for TMZ-resistant tumors [77]. Moreover, tinostamustine (TINO), as a first-in-class alkylating deacetylase inhibitor (AK-DACi), showed significant therapeutic activity with suppression of tumor growth and prolongation of OS in orthotopic intra-brain models, associated with higher caspase-3 activation and reduced autophagy [92]. Additionally, cell-cycle regulators offer additional opportunities for targeted intervention. Navtemadlin exhibits IC_50_ values of 0.1–0.2 μM in TP53-wild-type cell lines and restores p53 activity by selectively disrupting the interaction between MDM2 and p53. This mechanism is particularly relevant to tumors retaining functional TP53, a characteristic historically associated with the classical GBM subtype. Navtemadlin also promotes oligodendrocyte-like differentiation and circumvents resistance related to mismatch repair deficiency [78]. Avasimibe, an inhibitor of acyl-CoA cholesterol acyltransferase-1 (ACAT-1), suppresses cell viability with an IC_50_ of 20.0 μM in U251 and U87MG cells, induces arrest at both the G0/G1 and G2/M checkpoints, and activates mitochondria-mediated apoptosis [79]. The repurposed anthelmintic flubendazole has an IC_50_ of 0.2 μM in U87MG and U251 cells. It induces G2/M-phase arrest through the p53/p21/cyclin B1 axis and initiates both caspase-dependent apoptosis and pyroptosis mediated by the NF-κB/NOD-like receptor family pyrin domain-containing 3 (NLRP3)/gasdermin D (GSDMD) pathway [80].

### 5.3. Remodeling the Tumor Microenvironment: Metabolic and Immune Regulation

Reprogramming the immunosuppressive and metabolically dysregulated GBM microenvironment may create opportunities for effective combination therapy. At an in vitro concentration of 1 mM, metformin activates the AMPK–SIRT2 axis and promotes H3K56 and H4K16 deacetylation at the CCR8 promoter, thereby repressing CCR8 expression in CD4^+^ T cells [81]. This epigenetic regulation reduces tumor-infiltrating Treg cells, restores CD8^+^ T-cell cytotoxicity, and potentiates the therapeutic efficacy of PD-1 blockade in GBM [81]. Metabolic strategies can also exploit tumor-cell susceptibility to oxidative stress. Artesunate, an antimalarial agent with an IC_50_ of 75.0 μM in U251 and U118 cells, cooperates with metformin to induce ROS accumulation. The combination alters AMPK/mTOR signaling and ultimately triggers autophagy-dependent apoptosis [82]. Chemokine modulation provides another means of reshaping the immune microenvironment. Duloxetine, a repurposed antidepressant, reduces S100 calcium-binding protein B (S100B) expression in glioma cells and suppresses C-C motif chemokine ligand 2 (CCL2) secretion at a concentration of 5 μM. This effect limits the recruitment of TAMs and myeloid-derived suppressor cells (MDSCs), while directing the remaining myeloid populations toward a tumoricidal M1-like phenotype [93]. EP26 combines intracellular oncogenic inhibition with blockade of extracellular immune escape and has been reported to be a first-in-class dual inhibitor of PD-L1 and EGFR. It has antiproliferative activity with IC_50_ values of 0.3–1.2 μM in U87MG, U251, GL261, and U87MG-EGFRvIII models. Mechanistically, EP26 inhibits EGFR kinase activity and interferes with PD-1/PD-L1 binding, thereby restoring T-cell-mediated antitumor cytotoxicity in the GBM microenvironment [83].

## 6. Immunomodulatory Effects of Natural and Synthetic Compounds on GBM

From an immunological perspective, GBM is widely regarded as a “cold” tumor because it contains few effector T cells and exhibits a strongly immunosuppressive TME enriched in tumor-supportive myeloid populations. This immune landscape represents a major mechanism underlying resistance to current immune checkpoint blockade (ICB) therapies [94]. Compared with conventional macroscopic interventions and biological macromolecules, small-molecule immunomodulators provide several potential advantages by enabling targeted reprogramming of the TME instead of depending exclusively on broad cytotoxic effects. These agents may strengthen endogenous antitumor immunity by disrupting immunosuppressive signaling networks and inducing the differentiation of GSCs, thereby reducing cellular reservoirs associated with tumor recurrence [95]. Their suitability for oral administration may also facilitate prolonged maintenance treatment. As summarized in Table 4, these molecules reshape the GBM microenvironment by regulating multiple immune-cell populations through distinct molecular mechanisms.

### 6.1. Reprogramming T-Cell and NK Cell Immunity

Effective antitumor immunity requires the restoration of cytotoxic lymphocyte function and the removal of barriers that prevent these cells from entering tumor tissue. Epigenetic regulation and receptor-directed inhibition provide complementary approaches to reversing these defects. Metformin induces this epigenetic modification, represses CCR8 transcription, and consequently restores the cytotoxic activity of CD8^+^ T cells [81,88]. Immune-checkpoint intervention must both reinvigorate exhausted T cells and facilitate the accumulation of CD4^+^ and CD8^+^ T-cell subsets within the tumor. EP26 addresses these requirements by simultaneously inhibiting the PD-1/PD-L1 checkpoint axis and the tumor-associated signaling driver EGFR [83]. CBD acts through a related immunoregulatory mechanism by suppressing the IDO checkpoint, increasing integrin alpha E (CD103) expression, strengthening T-cell responses, and reducing innate lymphoid-cell populations [55,97]. In addition to adaptive immune reactivation, stimulation of innate cytotoxic cells represents another important therapeutic strategy. Repurposing irinotecan, a conventional topoisomerase I inhibitor, enhances the tumor-killing activity of natural killer (NK) cells and thereby provides complementary support for antitumor immunity [98].

### 6.2. Re-Educating TAMs and Microglia

Myeloid cells account for a substantial proportion of the immune infiltrate in GBM and predominantly acquire a tumor-supportive M2-like state. Redirecting these populations toward a tumoricidal M1-like phenotype is therefore an important immunomodulatory effect shared by several natural and synthetic small molecules. Ginsenoside Rg3 promotes M2-to-M1 repolarization by suppressing the IL-6/IL-23/STAT3 signaling cascade [35]. Bijangi-Vishehsaraei et al. further showed that metabolic reprogramming can induce a comparable phenotypic transition. Specifically, SFN inhibits TrxR1, leading to ROS accumulation and increased expression of proinflammatory markers [60,61]. Rutin reduces the production of glioma-derived immunosuppressive mediators, including TGF-β, and independently induces a proinflammatory M1-like state in microglia [45]. Chemokine regulation provides another approach to myeloid-cell reprogramming. Duloxetine decreases S100B expression and subsequently limits the release of the chemoattractant CCL2. This signaling change reduces the recruitment of TAMs and MDSCs, while directing the remaining myeloid populations toward an M1-like tumoricidal phenotype [81,93].

**Figure 4:**
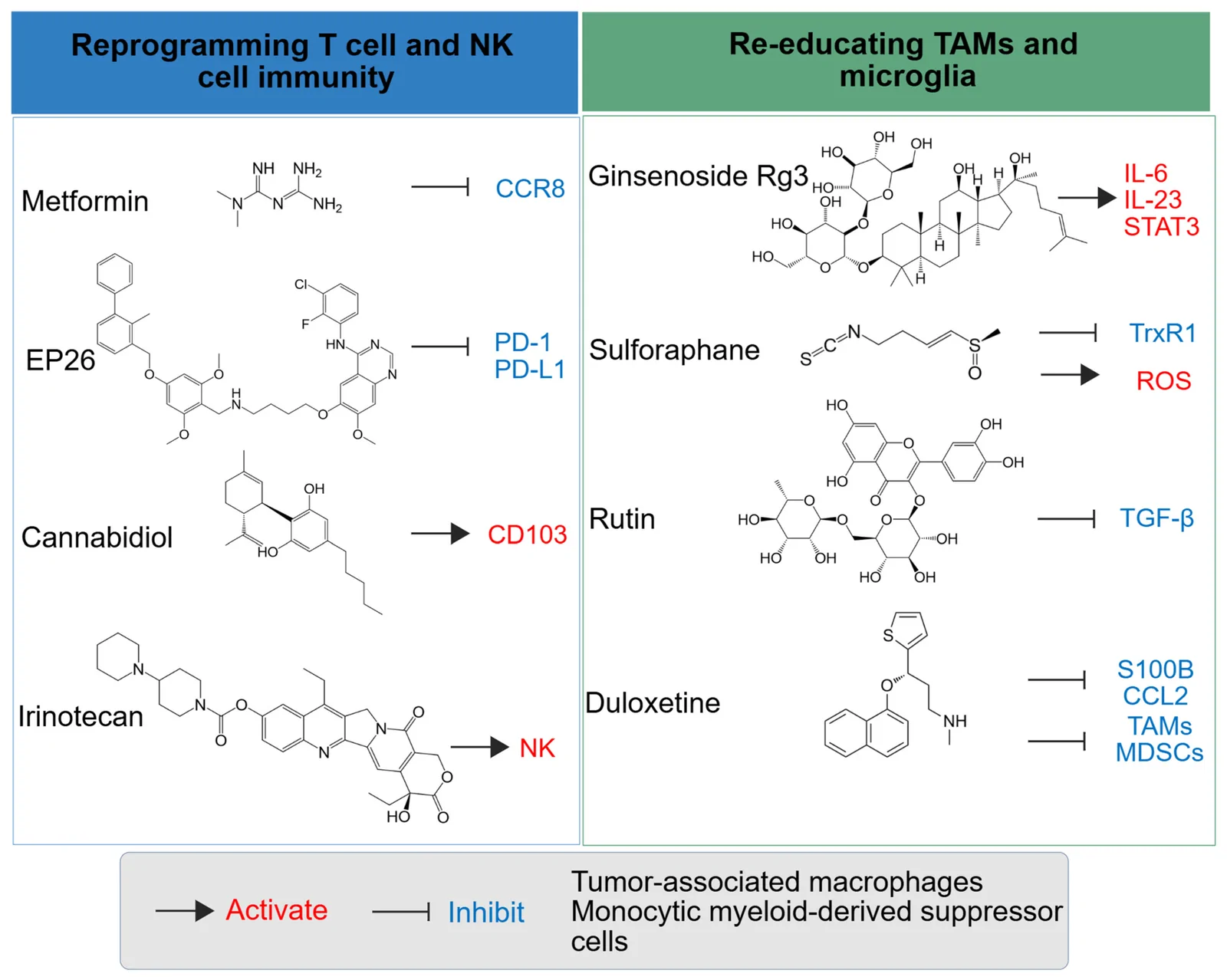
Immunomodulatory effects of natural and synthetic compounds against GBM. The figure summarizes strategies for reprogramming T-cell and NK-cell immunity and for re-educating TAMs and microglia.

In summary, these agents can coordinately regulate T cells, NK cells, and infiltrating myeloid populations. By attenuating immunosuppressive mechanisms and reprogramming tumor-supportive immune cells toward tumoricidal phenotypes, these compounds may promote the transition of GBM from an immunologically “cold” treatment-resistant malignancy into a more immune-responsive “hot” tumor. Such remodeling provides a biological rationale for developing next-generation combination immunotherapies (Figure 4).

## 7. Conclusions

This narrative review summarizes the molecular mechanisms and translational advances of natural and synthetic compounds for GBM treatment. These compounds suppress tumor progression and invasiveness and remodel the immunosuppressive tumor microenvironment, thereby providing an important strategy for overcoming resistance to current standard treatments and improving long-term outcomes for patients with GBM.

## 8. Challenges and Future Perspectives

Despite encouraging results from preclinical studies, the translation of small-molecule therapies into clinical practice for GBM continues to face substantial obstacles. To narrow the gap between experimental discovery and patient treatment, future investigations should systematically address the following strategic priorities:

### 8.1. Mining Other Vital Pathways or Targets

Beyond the dominant PI3K/Akt, STAT3, and NF-κB networks, the Wnt/β-catenin pathway, a conserved signaling cascade, has been recently shown to play a vital role in GBM progression by regulating glioma stem cell maintenance, tumor proliferation, invasion, and therapeutic resistance [86,99]. Physiologically, Wnt ligands bind Frizzled–LRP5/6 receptors to inhibit the Axin/APC/GSK-3β destruction complex, stabilizing β-catenin for nuclear transcription of proliferation and stemness genes. Tompa et al. demonstrated DNA methylation patterns of Wnt pathway components, including Wnt5a, Wnt3a, and β-catenin, in GBM samples [86]. That abnormal methylation-mediated regulation of Wnt pathway antagonists reduce endogenous pathway inhibition and promote persistent Wnt/β-catenin activation, highlighting the therapeutic significance of targeting Wnt-associated epigenetic alterations [86,99]. Curcumin has been shown to suppress GBM progression by inhibiting the AKT/Wnt/β-catenin signaling axis and reducing tumor growth in xenograft models [100], indicating that Wnt pathway modulation contributes to its anti-GBM activity and underscoring the importance of considering additional signaling pathways beyond the canonical networks.

### 8.2. Overcoming Druggable Bottlenecks

Favorable physicochemical properties may support BBB penetration, brain-to-plasma ratio, free drug concentration, efflux transport, tumor penetration, and target engagement, the lack of which contributes to the high failure rate of these therapeutic candidates. To overcome this challenge, novel cell-based evaluation models should be established. For natural compounds that have low solubility in aqueous media, rapid metabolic elimination, and insufficient systemic bioavailability in vivo, future studies should emphasize structure-guided medicinal chemistry optimization and the development of advanced nanocarrier platforms (lipid nanoparticles and targeted hydrogels) designed for CNS delivery [101,102,103].

### 8.3. Advanced Preclinical Models and Precision Medicine

The marked spatial and temporal heterogeneity of GBM produces substantial variation in treatment responsiveness and limits the clinical predictive value of conventional two-dimensional cell-line systems. Such models inadequately reproduce the complex TME, immune-cell infiltration, and stem-like properties observed in human GBM. Therefore, future preclinical research should make greater use of patient-derived organoids and sophisticated orthotopic patient-derived xenograft models [87,104,105]. Moreover, data mining and integrative bioinformatics approaches offer a powerful, cost-effective solution. Network pharmacology, molecular docking, and machine learning-based virtual screening can rapidly predict bioactive compounds, infer their molecular targets, and prioritize candidates for experimental validation. By mining publicly available transcriptomic, proteomic and chemical databases, compounds that simultaneously modulate multiple GBM-associated pathways should be identified [106], which could reduce the gap between future preclinical research and clinical use. Additionally, clinical management must progress beyond uniform treatment strategies toward individualized precision medicine. Rational regimens should integrate small-molecule agents with established interventions—including surgery, radiotherapy, TMZ, and immunotherapy—according to detailed molecular stratification. Relevant biomarkers include MGMT promoter methylation, IDH mutation status, and EGFR amplification. This individualized approach may improve therapeutic synergy and facilitate more durable tumor control.

## Figures and Tables

**Table 1:** Natural compounds with anti-GBM activity and their mechanisms of action.

Compounds	MW (g/mol)	Cell Line	IC_50_ (μM)/Exposure Time	Animal Model	In Vivo (mg/kg)	Primary Mechanism	Study Stage	Ref.
Ginsenoside Rg3 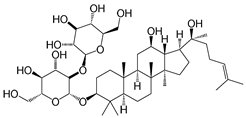	785.0	C6	35.0 (48 h)	Orthotopic C6 glioma-bearing model	20	Inhibits IL-6/IL-23/STAT3 axis, promotes M2→M1 polarization, enhances T-cell infiltration.	In vitro and in vivo	[35]
Luteolin 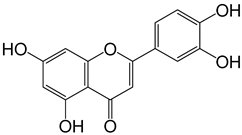	286.2	GSCs	50.0 (72 h)	None	NR	Inhibits RAS/MEK/ERK and PI3K/AKT/mTOR pathways; downregulates MGMT.	In vitro	[36]
Resveratrol 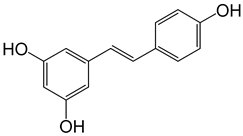	228.2	U87MG	20.0 (120 h)	U87MG xenograft model	50	Regulates AKT/p53/Nanog axis; inhibits proliferation/invasion, induces GSC differentiation.	In vitro and in vivo	[37,38]
Catechin 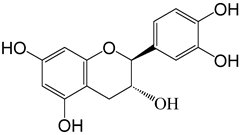	290.3	U87MG	20.0 (NR)	None	NR	Inhibits the MAPK pathway and modulates TNF-α expression.	In vitro	[39,40]
Polydatin 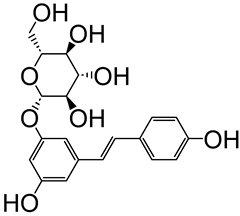	390.4	U87MG U251	440 (48 h) 256 (48 h)	Subcutaneous U251 xenograft model	50	Inhibits EGFR and downstream AKT/ERK/STAT3 pathways, downregulates SOX2/snail.	In vitro and in vivo	[41]
Icariside II 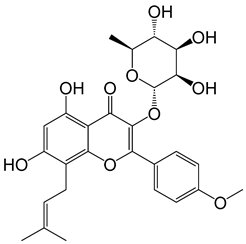	514.5	U87MG A172	30.0 (24 h) 35.0 (24 h)	None	NR	Inhibits AKT, promotes FOXO3a nuclear localization, upregulates p21/p27, G1 arrest.	In vitro	[42]
Baicalein 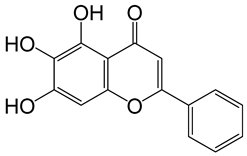	270.2	U251	30.0 (NR)	None	NR	Inhibits NF-κB p65 translocation, modulates BCL-2/BAX ratio, induces apoptosis.	In vitro	[43]
Apigenin 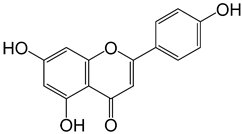	270.2	U87MG	~74.0 (48 h)	None	NR	Upregulates miR-16, suppresses BCL2 and NF-κB/MMP-9 signaling axes.	In vitro	[44]
Rutin 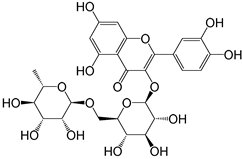	610.5	U251	50.0 (24 h)	Orthotopic U251 xenograft model	NR	Induces microglial M1 polarization and remodels the immune microenvironment.	In vitro and in vivo	[45]
Quercetin 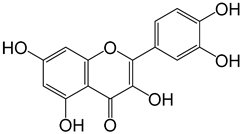	302.2	T98G	55.0 (48 h)	None	NR	Dual inhibition of Wnt3a/β-catenin and AKT/NF-κB pathways; downregulates MGMT.	In vitro	[46,47]
Parthenolide 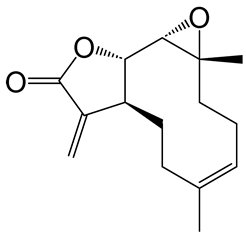	248.3	U87MG U373	5.0 (24 h) 5.0 (24 h)	Orthotopic U87MG xenograft model	10	Inhibits IκB kinase, blocks NF-κB p65; suppresses angiogenesis and GSC self-renewal.	In vitro and in vivo	[48,49,50]
Alantolactone 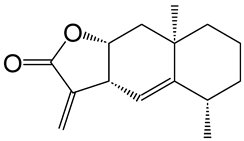	232.3	U87MG U251	20.0 (48 h) 16.0 (48 h)	Subcutaneous U87MG xenograft model	10 and 20.0	Targets IKKβ/NF-κB/COX-2 axis; induces cycle arrest/apoptosis, inhibits migration/invasion.	In vitro and in vivo	[51]
Molephantin 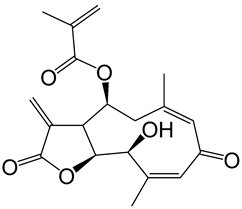	302.2	U87MG U251	23.0 (72 h) 11.0 (72 h)	Subcutaneous U87MG xenograft model	10 and 30	Induces ROS-mediated inhibition of the PI3K/AKT/mTOR pathway and apoptosis.	In vitro and in vivo	[52]
Forsythoside B 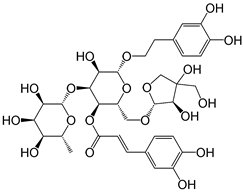	624.6	U251 C6	200 (48 h) 200 (72 h)	Subcutaneous U251 xenograft model (nude mice)	50	Upregulates PTPRN, induces G0/G1 arrest and apoptosis, inhibits migration.	In vitro and in vivo	[53]
Berberine 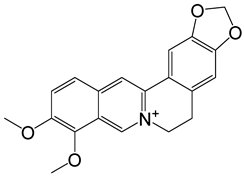	336.4	U87MG	25.0 (72 h)	None	NR	Induces oxidative stress-mediated apoptosis and arrests the cell cycle.	In vitro	[54]
Cannabidiol 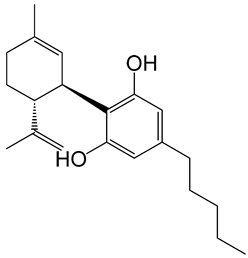	315.0	GSC 3832 GSC 387	3.5 μM (NR) 2.6 μM (NR)	Orthotopic GL261 syngeneic GBM model	10	Induces mitophagy, blocks IDO, and promotes T-cell response.	In vitro and in vivo	[55,56]
Tanshinone IIA 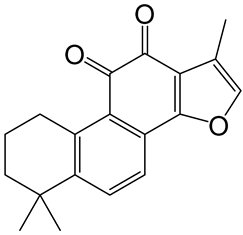	294.3	U87MG U251	10.0 (24–72 h)	U87MG xenograft nude mice	10.0 μM	Upregulates DR4/DR5, inhibits STAT3, activates caspases, and sensitizes to TRAIL.	In vitro and in vivo	[57]
Paeoniflorin 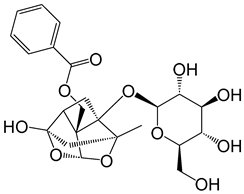	480.5	U87MG U251	5.0–10.0 (24 h)	Subcutaneous U87MG xenograft model	1000	Downregulates TGF-β expression, inhibits EMT, and promotes apoptosis.	In vitro and in vivo	[58]
Ferulic Acid 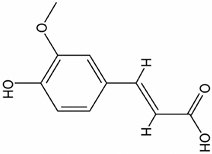	194.2	U87MG	36.0 (24 h)	None	NR	Regulates TG2 activity, blocks the cell cycle, and activates caspase-3/PARP-1.	In vitro	[59]
Sulforaphane 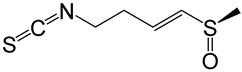	177.3	U87MG	35.0 (48 h)	GBM10 xenograft model (NSG mice)	100	Targets TrxR1 to induce ROS, activates apoptosis, drives M1 polarization, and sensitizes to chemoradiotherapy.	In vitro and in vivo	[60,61]
Bufotalin 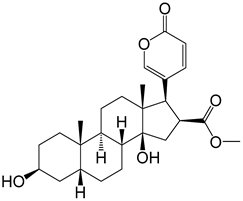	444.6	U87MG U251	0.1 (24 h) 0.2 (24 h)	Subcutaneous U87MG xenograft model	NR	Upregulates BAD, downregulates BCL-2, activates caspase-3, and induces apoptosis.	In vitro and in vivo	[62]
Curcumin 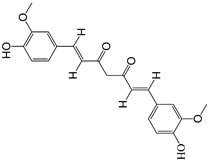	368.38	T98G U87MG	31.0 (24 h) 15.0 (24 h)	Orthotopic U87MG-luc xenograft model	60	Inhibits SHH/GLI1, PI3K/AKT, NF-κB and STAT3 signaling.	In vitro and in vivo	[63,64]

Abbreviations: NR, not reported in the original study.

**Table 2:** Clinical trial of natural and synthetic products for GBM.

Compound	Highest Clinical Phase in GBM	Trial Status	Disease Context	Trial Registration (No.)
Trametinib	Phase II	Active, not recruiting	NF1-mutant advanced cancers, including recurrent/progressive WHO grade 4 glioma (glioblastoma)	NCT04439318
Everolimus	Phase II	Completed	Newly diagnosed glioblastoma; everolimus evaluated in combination with radiotherapy and temozolomide	NCT01062399
Metformin	Phase II	Recruiting	IDH-wild-type glioblastoma; metformin investigated as an adjunct to temozolomide-based therapy	NCT05929495
Dichloroacetate (DCA)	Phase II (Phase IIA)	Active, not recruiting	Recurrent glioblastoma undergoing clinically indicated debulking surgery; evaluation of tumor pyruvate dehydrogenase complex phosphorylation	NCT05120284

**Table 3:** Synthetic compounds with anti-GBM activity and their mechanisms of action.

Compounds	MW (g/mol)	Cell Line	IC_50_ (μM)/Exposure Time	Animal Model	In Vivo (mg/kg)	Primary Mechanism	Study Stage	Ref.
Gefitinib 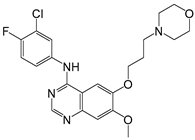	446.9	U87MG U373	20.0 (48 h) 20.0 (48 h)	U87MG xenograft SCID mice	50	Inhibits EGFR activity.	In vitro and in vivo	[73]
Osimertinib 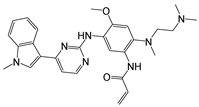	499.6	LN-18 LN-229 U87MG SF-539	4.0 (24 h)	Orthotopic intracranial GBM xenograft model	50	Induces ER stress-mediated paraptosis-like cell death.	In vitro and in vivo	[74]
Trametinib 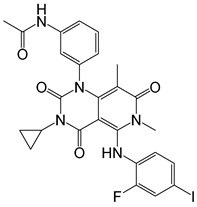	615.4	GSC	0.2 (72 h)	Orthotopic intracranial NPE-FAK-WT GBM xenograft model	0.5	Selectively inhibits MEK1/2; blocks MAPK/ERK pathway.	In vitro and in vivo	[75]
Foretinib 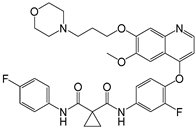	632.7	T98G U251 U87MG	4.7 (48 h) 22.4 (48 h) 30.0 (48 h)	None	NR	Induces G2/M arrest and mitochondria-dependent apoptosis.	In vitro	[76]
Everolimus 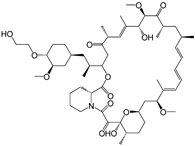	958.2	GBM PDX	NR	GBM PDX mouse model	5	mTORC1 inhibitor; inhibits the PI3K/AKT/mTOR pathway.	In vivo	[11]
Lomustine 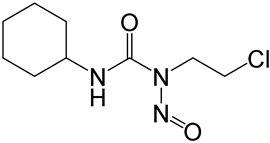	233.7	U87MG U87-R U251 U251-R U343 U343-R GS-Y03	55.0 (72 h) 86.0 (72 h) 44.0 (72 h) 48.0 (72 h) 96.0 (72 h) 71.0 (72 h) 12.0 (72 h)	Orthotopic intracranial U87MG/U87-R xenograft nude mouse model	20	Induces DNA alkylation, crosslinks, and double-strand-break signaling.	In vitro and in vivo	[77]
Nimustine 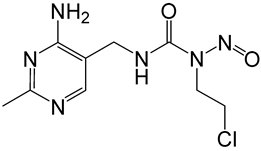	309.2	U87MG U87-R U251 U251-R U343 U343-R GS-Y03	262.0 (72 h) 283.0 (72 h) 310.0 (72 h) 293.0 (72 h) 281.0 (72 h) 295.0 (72 h) 406.0 (72 h)	Orthotopic intracranial U87MG/U87-R xenograft nude mouse model	15	Induces DNA alkylation-mediated damage and apoptotic signaling.	In vitro and in vivo	[77]
Navtemadlin 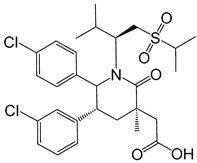	568.6	BT145 BT286 BT359	0.05 (72 h)	None	120 and 240	Selectively inhibits MDM2-p53 interaction; activates the p53 pathway.	In vitro and in vivo	[78]
Avasimibe 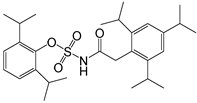	501.7	U251 U87MG	20.3 (48 h) 28.3 (48 h)	U87MG xenograft nude mice	15 and 30	Induces dual G0/G1 and G2/M arrest; triggers apoptosis.	In vitro and in vivo	[79]
Flubendazole 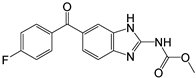	313.3	U87MG U251	0.2 (24 h) 0.2 (24 h)	U87MG xenograft nude mouse model	12.5, 25, 50	Induces G2/M arrest; inhibits EMT and stemness properties.	In vitro and in vivo	[80]
Metformin 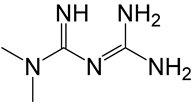	129.2	GL261	1000 (48 h)	Orthotopic GL261 glioblastoma model	50.0	Inhibits CCR8 expression; reduces Treg.	In vitro and in vivo	[81]
Artesunate 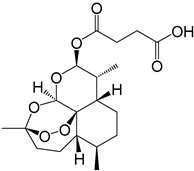	384.4	U251 U118	75.0 (48 h)	None	NR	Generates ROS, activates AMPK/mTOR axis; induces autophagy.	In vitro	[82]
EP26 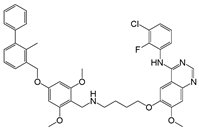	737.3	U87MG U251 GL261 U87MG-FRvIII	0.8 (72 h) 1.0 (72 h) 0.3 (72 h) 1.2 (72 h)	GL261 GBM-bearing C57BL/6 mice	100	Dual targeting of PD-L1/EGFR restores T-cell immune killing.	In vitro and in vivo	[83]
Palbociclib 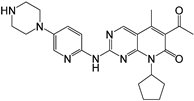	447.5	TMZ-resistant GBM TMZ-sensitive GBM cells	1.0 (NR) 0.5 (NR)	Patient-derived TMZ-resistant GBM xenograft model in NOD/SCID female mice	75	Inhibits CDK4/6; regulates lncRNA SNHG15/miR-627-5p axis.	In vitro and in vivo	[84]
Tinostamustine 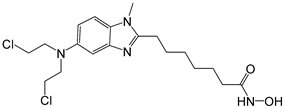	415.4	U87MG U251 T98G	52.0 (72 h)	U87MG/U251 xenograft; orthotopic U251 and CSCs-5 models	80–100 mg/m^2^	Dual alkylating and HDAC inhibition; increases DNA damage (γH2AX).	In vitro and in vivo	[85,86]
Irinotecan 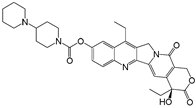	586.7	U87MG U118 U343	136.0 (48 h) 153.0 (48 h) 187.0 (48 h)	U87MG xenograft	125 mg/m^2^ or 60 mg/m^2^	Topoisomerase I inhibitor; enhances NK cell cytotoxicity.	In vitro and in vivo	[87]
Dichloroacetate 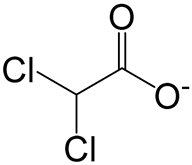	127.9	U251	~1500 (72 h)	U87MG, patient-derived GBM neurospheres	7.5	Inhibits (PDK1); promotes metabolic shift.	In vitro and in vivo	[88]

**Table 4:** Molecular mechanisms of natural and synthetic compounds regulating the GBM immune microenvironment.

Compound	Target/Pathway	Immune Cell Effect	Molecular Mechanism	Ref.
Metformin	AMPK/SIRT2/CCR8 axis	↓ Tregs, ↑ CD8^+^ T cells	CCR8 promoter histone deacetylation	[81]
Ginsenoside Rg3	IL-6/IL-23/STAT3	M2 → M1 polarization, ↑ CD8^+^ T cells	Inhibition of STAT3 phosphorylation	[35]
Sulforaphane	TrxR1-ROS	M2 → M1 polarization	ROS-mediated metabolic reprogramming	[60,61]
Duloxetine	S100B-CCL2	↓ TAM, ↓ MDSCs infiltration	Norepinephrine transporter inhibition	[93]
EP26	PD-L1/EGFR	Restores T-cell killing function	Dual-target inhibition	[83]
Cannabidiol	IDO, PD-L1	↑ CD8^+^ T-cell response	ICB	[55]
Ganoderma lucidum polysaccharides (GLPs)	IL-2R, TNFR, IFNGR	CD8^+^ T cells ↑, IL-2 ↑, TNF-α ↑	Enhancement of NK cell cytotoxicity	[96]
Rutin	IL-10, TGF-β	IL-1β, IL-18, NOS2, PTGS2 ↑	Remodeling of the immune microenvironment	[45]
Irinotecan	Topoisomerase I/DNA damage	↑ NK cell cytotoxicity	Induction of DNA damage and increased expression of NK-activating ligands (MICA/B)	[87]

Note: ↑, increased/upregulated; ↓, decreased/downregulated; →, phenotypic transition/polarization.

## Data Availability

No new data were created or analyzed in this study. Data sharing is not applicable to this article.
